# 4-(4-Chloro­phen­yl)-1-[3-(4-fluoro­benzo­yl)prop­yl]-4-hydroxy­piperidin-1-ium 2,4,6-trinitro­phenolate (haloperidol picrate)

**DOI:** 10.1107/S1600536809033261

**Published:** 2009-09-09

**Authors:** Jerry P. Jasinski, Ray J. Butcher, Q. N. M. Hakim Al-Arique, H. S. Yathirajan, B. Narayana

**Affiliations:** a Department of Chemistry, Keene State College, 229 Main Street, Keene, NH 03435-2001, USA; b Department of Chemistry, Howard University, 525 College Street NW, Washington, DC 20059, USA; c Department of Studies in Chemistry, University of Mysore, Manasagangotri, Mysore 570 006, India; d Department of Studies in Chemistry, Mangalore University, Mangalagangotri 574 199, India

## Abstract

In the title salt, C_21_H_24_ClFNO_2_^+^·C_6_H_2_N_3_O_7_^−^, the dihedral angle between the aromatic rings in the cation is 16.5 (1)°. The piperidium ring adopts a slightly distorted chair conformation. Strong hydrogen-bonding inter­actions occur between the N—H and O—H functions of the 4-hydroxy­piperidin-1-ium ring and the phenolate and *p*-NO_2_ O atoms of the picrate anion. In addition, a variety of weak C—H⋯O and π–π ring inter­actions between cations and cation–anion neighbors [centroid–centroid distances = 3.597 (1) and 3.848 (10) Å] further consolidate the packing.

## Related literature

For related structures, see: Casellato *et al.* (2003 ▶); Datta *et al.* (1979 ▶); Prasanna & Guru Row (2001 ▶); Reed & Schafer (1973 ▶). For general background, see: Kurzawa *et al.* (2004 ▶); Volavka & Cooper, (1987 ▶). For a description of the Cambridge Structural Database, see: Allen (2002 ▶) and for *Mogul*, see: Bruno *et al.* (2004 ▶). For puckering parameters, see: Cremer & Pople (1975 ▶).
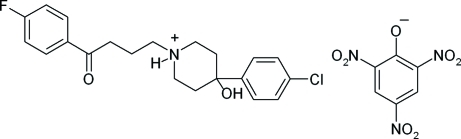


## Experimental

### 

#### Crystal data

C_21_H_24_ClFNO_2_^+^·C_6_H_2_N_3_O_7_^−^
*M* *_r_* = 604.97
Orthorhombic, 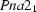

*a* = 14.9089 (5) Å
*b* = 12.5934 (3) Å
*c* = 14.5074 (5) Å
*V* = 2723.8 (2) Å^3^
*Z* = 4
Mo *K*α radiation
μ = 0.21 mm^−1^
*T* = 110 K
0.53 × 0.47 × 0.34 mm

#### Data collection

Oxford Diffraction Gemini R CCD diffractometer
Absorption correction: multi-scan (*CrysAlis RED*; Oxford Diffraction, 2007 ▶) *T*_min_ = 0.889, *T*_max_ = 0.931
19020 measured reflections
7472 independent reflections
5784 reflections with *I* > 2σ(*I*)
*R*_int_ = 0.034

#### Refinement

*R*[*F* ^2^ > 2σ(*F* ^2^)] = 0.037
*wR*(*F* ^2^) = 0.077
*S* = 0.92
7472 reflections
384 parameters
1 restraint
H-atom parameters constrained
Δρ_max_ = 0.30 e Å^−3^
Δρ_min_ = −0.24 e Å^−3^
Absolute structure: Flack (1983 ▶), 2287 Friedel pairs
Flack parameter: 0.03 (4)

### 

Data collection: *CrysAlis Pro* (Oxford Diffraction, 2007 ▶); cell refinement: *CrysAlis RED* (Oxford Diffraction, 2007 ▶); data reduction: *CrysAlis RED*; program(s) used to solve structure: *SHELXS97* (Sheldrick, 2008 ▶); program(s) used to refine structure: *SHELXL97* (Sheldrick, 2008 ▶); molecular graphics: *SHELXTL* (Sheldrick, 2008 ▶); software used to prepare material for publication: *SHELXTL*.

## Supplementary Material

Crystal structure: contains datablocks global, I. DOI: 10.1107/S1600536809033261/im2134sup1.cif

Structure factors: contains datablocks I. DOI: 10.1107/S1600536809033261/im2134Isup2.hkl

Additional supplementary materials:  crystallographic information; 3D view; checkCIF report

## Figures and Tables

**Table 1:** Hydrogen-bond geometry (Å, °)

*D*—H⋯*A*	*D*—H	H⋯*A*	*D*⋯*A*	*D*—H⋯*A*
O2*A*—H2*O*⋯O61*B*^i^	0.84	2.01	2.837 (2)	168
N1*A*—H1*N*⋯O1*B*	0.93	1.82	2.708 (1)	160
N1*A*—H1*N*⋯O62*B*	0.93	2.40	3.007 (2)	123
C3*A*—H3*AA*⋯O2*A*^ii^	0.95	2.47	3.338 (2)	152
C6*A*—H6*AA*⋯O22*B*^iii^	0.95	2.41	3.286 (2)	153
C8*A*—H8*AA*⋯O21*B*^iii^	0.99	2.61	3.544 (2)	158
C8*A*—H8*AB*⋯O61*B*^iv^	0.99	2.48	3.460 (2)	170
C14*A*—H14*A*⋯O62*B*^i^	0.99	2.59	3.486 (2)	150
C15*A*—H15*A*⋯O41*B*^v^	0.99	2.58	3.461 (2)	148
C18*A*—H18*A*⋯O22*B*^vi^	0.95	2.42	3.131 (2)	131

Symmetry codes: (i) 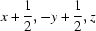; (ii) 
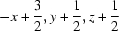
; (iii) 
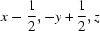
; (iv) 
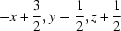
; (v) 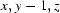; (vi) 
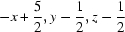
.

**Fig. 1.**
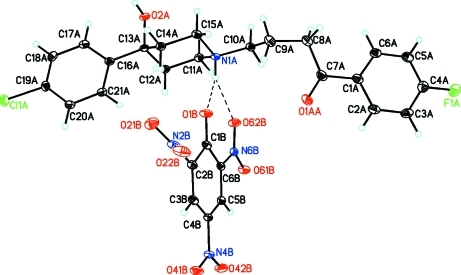
Molecular structure of the C21H24NO2ClF+. C6H2N3O7- cation-anion pair showing the atom labeling scheme and 50% probability displacement ellipsoids. Dashed lines indicate N1A–H1N···O1B and N1A–H1N···O62B hydrogen bond interactions.

**Fig. 2.**
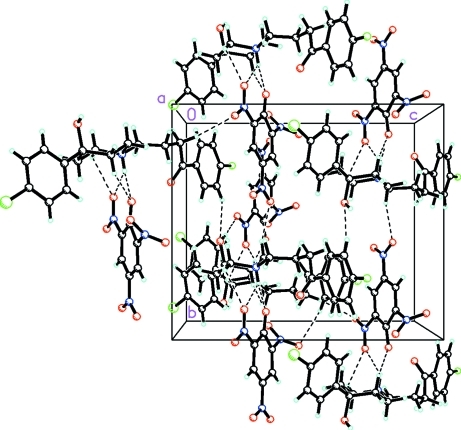
Packing diagram of the title compound, (I), viewed down the a axis. Dashed lines indicate intermolecular N–H···O & C–H···O hydrogen bond interactions which produces a network of infinite O–H···O–H···O–H chains arranged along the (011) plane of the unit cell.

## supplementary crystallographic information

### Comment

Haloperidol (IUPAC name: 4-[4-(4-Chlorophenyl)-4-hydroxy-1-piperidyl] -1-(4-fluorophenyl)-butan-1-one) is a typical antipsychotic and neuroleptic drug. It is in the butyrophenone class of antipsychotic medications and has pharmacological effects similar to the phenothiazines. Haloperidol possesses a strong activity against delusions and hallucinations, most likely due to an effective dopaminergic receptor blockage in the mesocortex and the limbic system of the brain. It blocks the dopaminergic action in the nigrostriatal pathways, which is the probable reason for the high frequency of extrapyramidal-motoric side-effects (dystonias, akathisia, pseudoparkinsonism). It also has minor antihistaminic and anticholinergic properties, therefore cardiovascular and anticholinergic side-effects such as hypotension, dry mouth, constipation, *etc*., are seen quite infrequently, compared with less potent neuroleptics such as chlorpromazine. A comprehensive review of haloperidol has found it to be an effective agent in treatment of symptoms associated with schizophrenia (Volavka & Cooper, 1987). The conductometric and spectrophotometric determination of haloperidol is described (Kurzawa *et al.* 2004). The crystal and molecular structures of haloperidol (Reed & Schaefer, 1979), haloperidol hydrobromide (Datta *et al.* 1979), flunarizine and haloperidol (Prasanna & Guru Row, 2001) and an analogue of haloperidol (Casellato *et al.* 2003) have been reported. In view of the importance of haloperidol and to study the hydrogen bonding patterns in the title compound, (I), C_27_H_26_O_9_N_4_ClF, a crystal structure is reported.

The title compound,*C*_27_H_26_N_4_O_9_ClF, crystallizes with one independent cation-anion pair [C_21_H_24_NO_2_ClF^+^. C_6_H_2_N_3_O_7_^-^] in the asymmetric unit. The haloperidol cation contains two halogen substituted benzene rings whose mean planes are separated by 16.5 (1)° and a 6-membered, 4-hydroxy-1-piperidinium group which adopts a slightly distorted chair conformation (Cremer & Pople, 1975) with puckering parameters Q, θ and φ of 0.5747 (6) Å, 0.4 (2)° and 349 (12)°, respectively (Fig. 1). For an ideal chair θ has a value of 0 or 180°. The dihedral angles between the mean planes of the fluoro and chloro substituted benzene rings and the mean plane of the 1-piperidyl group are 87.8 (5)° and 84.1 (5)°, respectively. The keto oxygen atom is disordered (O1AB = 0.72 (2) & O1AA = 0.28 (2)) with the major component (O1AB) making a twist angle of 15.4 (1)° (C1A/C7A/O1AB/C8A) with the fluorophenyl group. In the picrate anion, the mean planes of the two *o*-NO_2_ groups are twisted by 1.7 (5)° and 50.6 (7)° with respect to the mean plane of the 6-membered benzene ring (Fig. 2). The *p*-NO_2_ oxygen atoms are coplanar with respect to the mean plane of the benzene ring. The difference in the twist angles of the mean planes of the two *o*-NO_2_ groups can be partially attributed to the influence of a collection of weak hydrogen bonded interactions with neighboring cations (C8A–H8AA···O21B, C6A–H6AA···O22B, C18A–H18A···O22B) and with strong intermolecular "side" hydrogen bonds (N1A–H1N···O1B & N1A–H1N···O62B) with N1B from the 1-piperidinium group (Fig. 2, Table 1). H1N forms a bifurcated (three-center) hydrogen bond in this environment. Bond lengths and angles in both the cation and anion can be regarded as normal (Cambridge Structural Database, Version 5.30, February, 2009; Allen, 2002, *Mogul*, Version 1.1.3; Bruno *et al.*, 2004). Crystal packing is also influenced by additional weak π–π ring intermolecular interactions (*Cg*2···*Cg*4 = 3.597 (1) Å; 3/2 - *x*, -1/2 + *y*, 1/2 + *z*, and *Cg*3···*Cg*4 = 3.848 (10) Å; 2 - *x*, 1 - *y*, -1/2 + *z*, where *Cg*2 = C1A–C6A; C3g = C16A–C21A; C4g = C1B–C6B centroids).

### Experimental

Haloperidol (3.7 g, 0.01 mol) in 25 ml of methanol and picric acid (4.7 g, 0.01 mol) in 25 ml of methanol were mixed and stirred in a beaker at 318 K for two hours. The mixture was kept aside for about a week at room temperature. The separated salt was filtered, washed thoroughly with chloroform and dried in a vacuum desiccator over phosphorous pentoxide. The salt was recrystallized from *N*,*N*-dimethylformamide (m.p: 413- 416 K) by slow evaporation of the solvent.

### Refinement

The hydroxyl hydrogen atom (H20) was located in a Fourier map, and fixed at 0.84 Å. The rest of the H atoms were placed in their calculated positions and then refined using the riding model with O—H = 0.84, N—H = 0.93, C—H = 0.95–0.99 Å, and with *U*_iso_(H) = 1.17–1.49*U*_eq_(C,*O*,*N*).

### Crystal data

**Table:**

C_21_H_24_ClFNO_2_^+^·C_6_H_2_N_3_O_7_^−^	*F*(000) = 1256
*M**_r_* = 604.97	*D*_x_ = 1.475 Mg m^−^^3^
Orthorhombic, *P**n**a*2_1_	Mo *K*α radiation, λ = 0.71073 Å
Hall symbol: P 2c -2n	Cell parameters from 8346 reflections
*a* = 14.9089 (5) Å	θ = 5.0–32.6°
*b* = 12.5934 (3) Å	µ = 0.21 mm^−^^1^
*c* = 14.5074 (5) Å	*T* = 110 K
*V* = 2723.8 (2) Å^3^	Chunk, pale yellow
*Z* = 4	0.53 × 0.47 × 0.34 mm

### Data collection

**Table:**

Oxford Diffraction Gemini R CCD diffractometer	7472 independent reflections
Radiation source: Enhance (Mo) X-ray Source	5784 reflections with *I* > 2σ(*I*)
graphite	*R*_int_ = 0.034
Detector resolution: 10.5081 pixels mm^-1^	θ_max_ = 32.7°, θ_min_ = 5.0°
φ and ω scans	*h* = −21→22
Absorption correction: multi-scan (*CrysAlis RED*; Oxford Diffraction, 2007)	*k* = −11→18
*T*_min_ = 0.889, *T*_max_ = 0.931	*l* = −15→21
19020 measured reflections	

### Refinement

**Table:**

Refinement on *F*^2^	Secondary atom site location: difference Fourier map
Least-squares matrix: full	Hydrogen site location: inferred from neighbouring sites
*R*[*F*^2^ > 2σ(*F*^2^)] = 0.037	H-atom parameters constrained
*wR*(*F*^2^) = 0.077	*w* = 1/[σ^2^(*F*_o_^2^) + (0.0419*P*)^2^] where *P* = (*F*_o_^2^ + 2*F*_c_^2^)/3
*S* = 0.92	(Δ/σ)_max_ = 0.002
7472 reflections	Δρ_max_ = 0.30 e Å^−^^3^
384 parameters	Δρ_min_ = −0.24 e Å^−^^3^
1 restraint	Absolute structure: Flack (1983), 2287 Friedel pairs
Primary atom site location: structure-invariant direct methods	Flack parameter: 0.03 (4)

### Special details

**Table:**

Geometry. All e.s.d.'s (except the e.s.d. in the dihedral angle between two l.s. planes) are estimated using the full covariance matrix. The cell e.s.d.'s are taken into account individually in the estimation of e.s.d.'s in distances, angles and torsion angles; correlations between e.s.d.'s in cell parameters are only used when they are defined by crystal symmetry. An approximate (isotropic) treatment of cell e.s.d.'s is used for estimating e.s.d.'s involving l.s. planes.
Refinement. Refinement of *F*^2^ against ALL reflections. The weighted *R*-factor *wR* and goodness of fit *S* are based on *F*^2^, conventional *R*-factors *R* are based on *F*, with *F* set to zero for negative *F*^2^. The threshold expression of *F*^2^ > σ(*F*^2^) is used only for calculating *R*-factors(gt) *etc*. and is not relevant to the choice of reflections for refinement. *R*-factors based on *F*^2^ are statistically about twice as large as those based on *F*, and *R*- factors based on ALL data will be even larger.

### Fractional atomic coordinates and isotropic or equivalent isotropic displacement parameters (Å^2^)

**Table:**

	*x*	*y*	*z*	*U*_iso_*/*U*_eq_	Occ. (<1)
Cl1A	1.34757 (3)	0.42144 (3)	0.45280 (3)	0.03220 (10)	
F1A	0.37134 (7)	0.23216 (9)	1.20789 (10)	0.0486 (3)	
O1AA	0.7069 (12)	0.3214 (11)	0.9777 (13)	0.0512 (15)	0.28 (2)
O1AB	0.7328 (7)	0.3329 (4)	0.9962 (6)	0.0512 (15)	0.72 (2)
O2A	1.06195 (7)	0.08904 (7)	0.66072 (9)	0.0258 (3)	
H2O	1.1099	0.0624	0.6805	0.031*	
N1A	0.90790 (8)	0.20672 (9)	0.80712 (9)	0.0186 (3)	
H1N	0.9070	0.2798	0.8163	0.022*	
C1A	0.61183 (12)	0.24014 (12)	1.07122 (12)	0.0276 (4)	
C2A	0.57077 (13)	0.33404 (13)	1.10171 (13)	0.0310 (4)	
H2AA	0.5990	0.4003	1.0900	0.037*	
C3A	0.49075 (13)	0.33187 (13)	1.14801 (13)	0.0322 (4)	
H3AA	0.4639	0.3956	1.1696	0.039*	
C4A	0.45020 (12)	0.23523 (14)	1.16256 (13)	0.0321 (4)	
C5A	0.48711 (12)	0.14068 (13)	1.13194 (13)	0.0316 (4)	
H5AA	0.4568	0.0752	1.1413	0.038*	
C6A	0.56849 (12)	0.14369 (12)	1.08783 (12)	0.0284 (4)	
H6AA	0.5957	0.0793	1.0683	0.034*	
C7A	0.70033 (13)	0.24665 (12)	1.02276 (14)	0.0337 (4)	
C8A	0.75704 (12)	0.14780 (12)	1.01555 (12)	0.0282 (4)	
H8AA	0.7222	0.0920	0.9836	0.034*	
H8AB	0.7707	0.1218	1.0784	0.034*	
C9A	0.84472 (12)	0.16547 (13)	0.96399 (13)	0.0292 (4)	
H9AA	0.8695	0.2359	0.9806	0.035*	
H9AB	0.8887	0.1108	0.9828	0.035*	
C10A	0.83088 (10)	0.15999 (11)	0.86049 (11)	0.0207 (3)	
H10A	0.8230	0.0849	0.8421	0.025*	
H10B	0.7752	0.1986	0.8443	0.025*	
C11A	0.89490 (10)	0.18710 (12)	0.70629 (11)	0.0206 (3)	
H11A	0.8359	0.2155	0.6870	0.025*	
H11B	0.8951	0.1097	0.6945	0.025*	
C12A	0.96782 (10)	0.23902 (11)	0.64998 (12)	0.0219 (3)	
H12A	0.9642	0.3170	0.6576	0.026*	
H12B	0.9583	0.2226	0.5840	0.026*	
C13A	1.06140 (10)	0.20075 (10)	0.67912 (12)	0.0206 (3)	
C14A	1.07294 (10)	0.21878 (11)	0.78277 (12)	0.0220 (3)	
H14A	1.1314	0.1894	0.8026	0.026*	
H14B	1.0733	0.2960	0.7955	0.026*	
C15A	0.99821 (10)	0.16667 (11)	0.83823 (12)	0.0218 (3)	
H15A	1.0010	0.0887	0.8302	0.026*	
H15B	1.0065	0.1826	0.9045	0.026*	
C16A	1.13368 (10)	0.25698 (11)	0.62300 (12)	0.0216 (3)	
C17A	1.18453 (11)	0.20234 (12)	0.55825 (12)	0.0240 (3)	
H17A	1.1747	0.1285	0.5497	0.029*	
C18A	1.24937 (11)	0.25346 (12)	0.50584 (12)	0.0254 (3)	
H18A	1.2838	0.2148	0.4621	0.031*	
C19A	1.26326 (10)	0.36054 (12)	0.51775 (12)	0.0240 (3)	
C20A	1.21366 (11)	0.41814 (12)	0.58018 (13)	0.0272 (4)	
H20A	1.2231	0.4924	0.5869	0.033*	
C21A	1.14966 (11)	0.36643 (12)	0.63330 (12)	0.0257 (3)	
H21A	1.1161	0.4056	0.6774	0.031*	
O1B	0.94487 (8)	0.41234 (7)	0.84639 (9)	0.0295 (3)	
O21B	1.11786 (8)	0.49095 (10)	0.85835 (11)	0.0404 (3)	
O22B	1.09827 (10)	0.58450 (9)	0.98236 (10)	0.0400 (3)	
O41B	0.93213 (9)	0.90496 (8)	0.86429 (10)	0.0362 (3)	
O42B	0.80467 (8)	0.87709 (8)	0.79751 (11)	0.0382 (3)	
O61B	0.70990 (7)	0.52946 (8)	0.72810 (9)	0.0295 (3)	
O62B	0.78680 (7)	0.39063 (8)	0.76248 (10)	0.0334 (3)	
N2B	1.07400 (10)	0.55211 (10)	0.90649 (11)	0.0289 (3)	
N4B	0.87478 (9)	0.84545 (10)	0.83136 (10)	0.0262 (3)	
N6B	0.77742 (8)	0.48709 (9)	0.76187 (10)	0.0200 (3)	
C1B	0.92607 (10)	0.50846 (11)	0.83829 (11)	0.0198 (3)	
C2B	0.98785 (10)	0.58939 (11)	0.87141 (11)	0.0208 (3)	
C3B	0.97235 (11)	0.69557 (11)	0.87218 (11)	0.0222 (3)	
H3BA	1.0146	0.7435	0.8981	0.027*	
C4B	0.89221 (10)	0.73212 (11)	0.83366 (12)	0.0213 (3)	
C5B	0.82938 (10)	0.66318 (11)	0.79824 (11)	0.0197 (3)	
H5BA	0.7754	0.6896	0.7721	0.024*	
C6B	0.84571 (9)	0.55469 (11)	0.80103 (11)	0.0185 (3)	

### Atomic displacement parameters (Å^2^)

**Table:**

	*U*^11^	*U*^22^	*U*^33^	*U*^12^	*U*^13^	*U*^23^
Cl1A	0.02537 (19)	0.0366 (2)	0.0347 (2)	−0.00290 (16)	0.00370 (19)	0.0040 (2)
F1A	0.0313 (6)	0.0495 (6)	0.0650 (9)	0.0082 (5)	0.0082 (6)	0.0025 (6)
O1AA	0.062 (3)	0.0202 (11)	0.071 (3)	−0.0155 (15)	0.032 (3)	−0.0022 (14)
O1AB	0.062 (3)	0.0202 (11)	0.071 (3)	−0.0155 (15)	0.032 (3)	−0.0022 (14)
O2A	0.0220 (6)	0.0150 (4)	0.0403 (7)	0.0024 (4)	−0.0042 (5)	−0.0022 (5)
N1A	0.0202 (6)	0.0127 (5)	0.0229 (7)	−0.0005 (4)	−0.0043 (5)	0.0007 (5)
C1A	0.0413 (10)	0.0230 (7)	0.0186 (8)	−0.0022 (7)	−0.0016 (7)	0.0006 (6)
C2A	0.0464 (11)	0.0218 (7)	0.0249 (9)	0.0001 (7)	−0.0048 (8)	0.0017 (7)
C3A	0.0417 (10)	0.0265 (8)	0.0284 (10)	0.0108 (7)	−0.0082 (8)	0.0001 (7)
C4A	0.0271 (9)	0.0389 (9)	0.0303 (10)	0.0047 (7)	−0.0040 (8)	0.0024 (8)
C5A	0.0352 (10)	0.0258 (8)	0.0340 (10)	−0.0050 (7)	−0.0048 (8)	0.0010 (7)
C6A	0.0405 (10)	0.0204 (7)	0.0243 (9)	0.0009 (6)	−0.0032 (8)	−0.0035 (7)
C7A	0.0509 (11)	0.0232 (8)	0.0270 (9)	−0.0062 (7)	0.0094 (9)	−0.0016 (7)
C8A	0.0354 (9)	0.0255 (7)	0.0236 (9)	−0.0061 (6)	−0.0024 (8)	0.0028 (7)
C9A	0.0297 (8)	0.0319 (8)	0.0260 (9)	−0.0084 (7)	−0.0050 (7)	0.0039 (7)
C10A	0.0197 (7)	0.0176 (6)	0.0247 (8)	−0.0032 (5)	−0.0025 (6)	0.0008 (6)
C11A	0.0190 (7)	0.0211 (6)	0.0217 (8)	0.0017 (5)	−0.0049 (6)	−0.0005 (6)
C12A	0.0209 (8)	0.0209 (7)	0.0239 (8)	0.0033 (5)	−0.0040 (6)	0.0022 (6)
C13A	0.0191 (7)	0.0135 (6)	0.0292 (9)	0.0026 (5)	−0.0022 (6)	0.0012 (6)
C14A	0.0187 (7)	0.0175 (6)	0.0298 (9)	−0.0003 (5)	−0.0046 (6)	0.0021 (6)
C15A	0.0186 (7)	0.0195 (7)	0.0271 (8)	−0.0013 (5)	−0.0080 (6)	0.0046 (6)
C16A	0.0188 (7)	0.0185 (7)	0.0276 (8)	0.0019 (5)	−0.0045 (6)	0.0031 (6)
C17A	0.0246 (8)	0.0186 (7)	0.0286 (9)	0.0024 (6)	−0.0058 (7)	−0.0020 (6)
C18A	0.0224 (8)	0.0298 (8)	0.0241 (9)	0.0063 (6)	−0.0025 (7)	−0.0024 (7)
C19A	0.0170 (7)	0.0299 (7)	0.0250 (9)	−0.0003 (6)	−0.0023 (6)	0.0052 (7)
C20A	0.0263 (8)	0.0185 (7)	0.0368 (10)	0.0010 (6)	0.0016 (8)	0.0025 (7)
C21A	0.0256 (8)	0.0184 (7)	0.0331 (10)	0.0037 (6)	0.0020 (7)	−0.0003 (7)
O1B	0.0305 (6)	0.0142 (5)	0.0438 (8)	−0.0018 (4)	−0.0126 (6)	−0.0010 (5)
O21B	0.0274 (7)	0.0356 (7)	0.0582 (9)	0.0055 (5)	−0.0121 (6)	−0.0040 (7)
O22B	0.0523 (8)	0.0237 (6)	0.0439 (8)	−0.0080 (5)	−0.0284 (7)	0.0023 (6)
O41B	0.0394 (7)	0.0167 (5)	0.0525 (9)	−0.0058 (5)	−0.0035 (6)	−0.0052 (6)
O42B	0.0315 (7)	0.0203 (5)	0.0628 (9)	0.0053 (5)	−0.0052 (7)	−0.0002 (6)
O61B	0.0273 (6)	0.0221 (5)	0.0392 (7)	−0.0005 (4)	−0.0148 (5)	0.0015 (5)
O62B	0.0259 (6)	0.0161 (5)	0.0582 (9)	−0.0023 (4)	−0.0096 (6)	−0.0047 (5)
N2B	0.0296 (8)	0.0170 (6)	0.0401 (9)	−0.0064 (5)	−0.0144 (7)	0.0048 (6)
N4B	0.0314 (7)	0.0139 (6)	0.0334 (8)	−0.0014 (5)	0.0032 (6)	−0.0011 (6)
N6B	0.0203 (6)	0.0178 (6)	0.0220 (7)	−0.0027 (4)	0.0002 (5)	−0.0013 (5)
C1B	0.0244 (7)	0.0163 (6)	0.0188 (7)	−0.0040 (5)	−0.0019 (6)	0.0006 (6)
C2B	0.0223 (7)	0.0183 (6)	0.0220 (8)	−0.0022 (5)	−0.0040 (6)	0.0016 (6)
C3B	0.0277 (8)	0.0181 (7)	0.0209 (8)	−0.0064 (5)	−0.0022 (7)	−0.0009 (6)
C4B	0.0250 (8)	0.0124 (6)	0.0263 (8)	−0.0013 (5)	0.0037 (7)	−0.0013 (6)
C5B	0.0220 (7)	0.0177 (7)	0.0195 (7)	−0.0004 (5)	0.0018 (6)	−0.0001 (6)
C6B	0.0207 (7)	0.0158 (6)	0.0189 (7)	−0.0043 (5)	0.0006 (6)	−0.0002 (6)

### Geometric parameters (Å, °)

**Table:**

Cl1A—C19A	1.7482 (16)	C13A—C16A	1.525 (2)
F1A—C4A	1.348 (2)	C13A—C14A	1.530 (2)
O1AA—C7A	1.151 (15)	C14A—C15A	1.523 (2)
O1AB—C7A	1.250 (6)	C14A—H14A	0.9900
O2A—C13A	1.4320 (16)	C14A—H14B	0.9900
O2A—H2O	0.8400	C15A—H15A	0.9900
N1A—C11A	1.496 (2)	C15A—H15B	0.9900
N1A—C10A	1.5047 (19)	C16A—C17A	1.389 (2)
N1A—C15A	1.5070 (19)	C16A—C21A	1.407 (2)
N1A—H1N	0.9300	C17A—C18A	1.388 (2)
C1A—C6A	1.397 (2)	C17A—H17A	0.9500
C1A—C2A	1.403 (2)	C18A—C19A	1.375 (2)
C1A—C7A	1.497 (3)	C18A—H18A	0.9500
C2A—C3A	1.369 (3)	C19A—C20A	1.376 (2)
C2A—H2AA	0.9500	C20A—C21A	1.389 (2)
C3A—C4A	1.375 (2)	C20A—H20A	0.9500
C3A—H3AA	0.9500	C21A—H21A	0.9500
C4A—C5A	1.385 (2)	O1B—C1B	1.2481 (17)
C5A—C6A	1.372 (3)	O21B—N2B	1.228 (2)
C5A—H5AA	0.9500	O22B—N2B	1.2284 (19)
C6A—H6AA	0.9500	O41B—N4B	1.2333 (18)
C7A—C8A	1.508 (2)	O42B—N4B	1.2217 (18)
C8A—C9A	1.522 (2)	O61B—N6B	1.2403 (16)
C8A—H8AA	0.9900	O62B—N6B	1.2229 (15)
C8A—H8AB	0.9900	N2B—C2B	1.459 (2)
C9A—C10A	1.517 (2)	N4B—C4B	1.4511 (18)
C9A—H9AA	0.9900	N6B—C6B	1.4436 (18)
C9A—H9AB	0.9900	C1B—C6B	1.438 (2)
C10A—H10A	0.9900	C1B—C2B	1.455 (2)
C10A—H10B	0.9900	C2B—C3B	1.357 (2)
C11A—C12A	1.509 (2)	C3B—C4B	1.397 (2)
C11A—H11A	0.9900	C3B—H3BA	0.9500
C11A—H11B	0.9900	C4B—C5B	1.377 (2)
C12A—C13A	1.535 (2)	C5B—C6B	1.3883 (18)
C12A—H12A	0.9900	C5B—H5BA	0.9500
C12A—H12B	0.9900		
			
C13A—O2A—H2O	109.5	O2A—C13A—C14A	109.17 (12)
C11A—N1A—C10A	109.86 (11)	C16A—C13A—C14A	112.10 (12)
C11A—N1A—C15A	110.68 (12)	O2A—C13A—C12A	105.20 (11)
C10A—N1A—C15A	113.38 (11)	C16A—C13A—C12A	110.45 (12)
C11A—N1A—H1N	107.6	C14A—C13A—C12A	109.03 (13)
C10A—N1A—H1N	107.6	C15A—C14A—C13A	111.90 (12)
C15A—N1A—H1N	107.6	C15A—C14A—H14A	109.2
C6A—C1A—C2A	118.48 (17)	C13A—C14A—H14A	109.2
C6A—C1A—C7A	122.43 (15)	C15A—C14A—H14B	109.2
C2A—C1A—C7A	119.09 (15)	C13A—C14A—H14B	109.2
C3A—C2A—C1A	121.19 (16)	H14A—C14A—H14B	107.9
C3A—C2A—H2AA	119.4	N1A—C15A—C14A	110.56 (12)
C1A—C2A—H2AA	119.4	N1A—C15A—H15A	109.5
C2A—C3A—C4A	118.42 (16)	C14A—C15A—H15A	109.5
C2A—C3A—H3AA	120.8	N1A—C15A—H15B	109.5
C4A—C3A—H3AA	120.8	C14A—C15A—H15B	109.5
F1A—C4A—C3A	118.94 (16)	H15A—C15A—H15B	108.1
F1A—C4A—C5A	118.58 (16)	C17A—C16A—C21A	117.69 (14)
C3A—C4A—C5A	122.48 (17)	C17A—C16A—C13A	121.10 (13)
C6A—C5A—C4A	118.50 (16)	C21A—C16A—C13A	121.20 (14)
C6A—C5A—H5AA	120.8	C18A—C17A—C16A	121.37 (13)
C4A—C5A—H5AA	120.8	C18A—C17A—H17A	119.3
C5A—C6A—C1A	120.89 (16)	C16A—C17A—H17A	119.3
C5A—C6A—H6AA	119.6	C19A—C18A—C17A	119.40 (15)
C1A—C6A—H6AA	119.6	C19A—C18A—H18A	120.3
O1AA—C7A—O1AB	23.2 (8)	C17A—C18A—H18A	120.3
O1AA—C7A—C1A	112.8 (8)	C18A—C19A—C20A	121.24 (14)
O1AB—C7A—C1A	122.2 (3)	C18A—C19A—Cl1A	118.08 (12)
O1AA—C7A—C8A	126.0 (8)	C20A—C19A—Cl1A	120.66 (11)
O1AB—C7A—C8A	118.6 (3)	C19A—C20A—C21A	119.17 (13)
C1A—C7A—C8A	118.76 (14)	C19A—C20A—H20A	120.4
C7A—C8A—C9A	113.24 (13)	C21A—C20A—H20A	120.4
C7A—C8A—H8AA	108.9	C20A—C21A—C16A	121.13 (15)
C9A—C8A—H8AA	108.9	C20A—C21A—H21A	119.4
C7A—C8A—H8AB	108.9	C16A—C21A—H21A	119.4
C9A—C8A—H8AB	108.9	O21B—N2B—O22B	124.12 (15)
H8AA—C8A—H8AB	107.7	O21B—N2B—C2B	118.17 (14)
C10A—C9A—C8A	111.27 (14)	O22B—N2B—C2B	117.71 (15)
C10A—C9A—H9AA	109.4	O42B—N4B—O41B	123.42 (12)
C8A—C9A—H9AA	109.4	O42B—N4B—C4B	118.90 (13)
C10A—C9A—H9AB	109.4	O41B—N4B—C4B	117.68 (13)
C8A—C9A—H9AB	109.4	O62B—N6B—O61B	121.54 (12)
H9AA—C9A—H9AB	108.0	O62B—N6B—C6B	120.16 (12)
N1A—C10A—C9A	112.81 (12)	O61B—N6B—C6B	118.31 (11)
N1A—C10A—H10A	109.0	O1B—C1B—C6B	127.98 (13)
C9A—C10A—H10A	109.0	O1B—C1B—C2B	120.40 (14)
N1A—C10A—H10B	109.0	C6B—C1B—C2B	111.59 (12)
C9A—C10A—H10B	109.0	C3B—C2B—C1B	125.81 (14)
H10A—C10A—H10B	107.8	C3B—C2B—N2B	117.65 (13)
N1A—C11A—C12A	111.37 (12)	C1B—C2B—N2B	116.54 (12)
N1A—C11A—H11A	109.4	C2B—C3B—C4B	117.84 (13)
C12A—C11A—H11A	109.4	C2B—C3B—H3BA	121.1
N1A—C11A—H11B	109.4	C4B—C3B—H3BA	121.1
C12A—C11A—H11B	109.4	C5B—C4B—C3B	121.56 (13)
H11A—C11A—H11B	108.0	C5B—C4B—N4B	119.33 (14)
C11A—C12A—C13A	111.70 (12)	C3B—C4B—N4B	119.11 (13)
C11A—C12A—H12A	109.3	C4B—C5B—C6B	119.37 (14)
C13A—C12A—H12A	109.3	C4B—C5B—H5BA	120.3
C11A—C12A—H12B	109.3	C6B—C5B—H5BA	120.3
C13A—C12A—H12B	109.3	C5B—C6B—C1B	123.76 (13)
H12A—C12A—H12B	107.9	C5B—C6B—N6B	116.43 (13)
O2A—C13A—C16A	110.65 (12)	C1B—C6B—N6B	119.79 (12)
			
C6A—C1A—C2A—C3A	1.0 (3)	C12A—C13A—C16A—C21A	67.61 (19)
C7A—C1A—C2A—C3A	−178.65 (17)	C21A—C16A—C17A—C18A	0.4 (2)
C1A—C2A—C3A—C4A	−1.3 (3)	C13A—C16A—C17A—C18A	178.89 (14)
C2A—C3A—C4A—F1A	−179.87 (17)	C16A—C17A—C18A—C19A	−0.4 (2)
C2A—C3A—C4A—C5A	−0.2 (3)	C17A—C18A—C19A—C20A	−0.5 (2)
F1A—C4A—C5A—C6A	−178.39 (17)	C17A—C18A—C19A—Cl1A	178.28 (12)
C3A—C4A—C5A—C6A	2.0 (3)	C18A—C19A—C20A—C21A	1.3 (3)
C4A—C5A—C6A—C1A	−2.2 (3)	Cl1A—C19A—C20A—C21A	−177.43 (13)
C2A—C1A—C6A—C5A	0.8 (3)	C19A—C20A—C21A—C16A	−1.2 (3)
C7A—C1A—C6A—C5A	−179.57 (17)	C17A—C16A—C21A—C20A	0.4 (2)
C6A—C1A—C7A—O1AA	145.5 (10)	C13A—C16A—C21A—C20A	−178.05 (15)
C2A—C1A—C7A—O1AA	−34.8 (10)	O1B—C1B—C2B—C3B	−175.26 (16)
C6A—C1A—C7A—O1AB	169.5 (6)	C6B—C1B—C2B—C3B	2.9 (2)
C2A—C1A—C7A—O1AB	−10.9 (7)	O1B—C1B—C2B—N2B	4.9 (2)
C6A—C1A—C7A—C8A	−17.8 (3)	C6B—C1B—C2B—N2B	−176.93 (14)
C2A—C1A—C7A—C8A	161.86 (17)	O21B—N2B—C2B—C3B	−129.26 (17)
O1AA—C7A—C8A—C9A	18.6 (12)	O22B—N2B—C2B—C3B	50.4 (2)
O1AB—C7A—C8A—C9A	−7.5 (6)	O21B—N2B—C2B—C1B	50.6 (2)
C1A—C7A—C8A—C9A	179.51 (15)	O22B—N2B—C2B—C1B	−129.80 (15)
C7A—C8A—C9A—C10A	−82.40 (18)	C1B—C2B—C3B—C4B	−3.5 (3)
C11A—N1A—C10A—C9A	173.30 (12)	N2B—C2B—C3B—C4B	176.28 (15)
C15A—N1A—C10A—C9A	48.88 (16)	C2B—C3B—C4B—C5B	1.8 (2)
C8A—C9A—C10A—N1A	163.71 (12)	C2B—C3B—C4B—N4B	−178.45 (15)
C10A—N1A—C11A—C12A	176.32 (11)	O42B—N4B—C4B—C5B	−0.4 (2)
C15A—N1A—C11A—C12A	−57.71 (14)	O41B—N4B—C4B—C5B	179.46 (16)
N1A—C11A—C12A—C13A	57.12 (16)	O42B—N4B—C4B—C3B	179.91 (16)
C11A—C12A—C13A—O2A	62.24 (16)	O41B—N4B—C4B—C3B	−0.3 (2)
C11A—C12A—C13A—C16A	−178.33 (13)	C3B—C4B—C5B—C6B	0.2 (2)
C11A—C12A—C13A—C14A	−54.73 (16)	N4B—C4B—C5B—C6B	−179.53 (13)
O2A—C13A—C14A—C15A	−59.63 (15)	C4B—C5B—C6B—C1B	−0.8 (2)
C16A—C13A—C14A—C15A	177.41 (11)	C4B—C5B—C6B—N6B	−179.40 (15)
C12A—C13A—C14A—C15A	54.80 (15)	O1B—C1B—C6B—C5B	177.33 (16)
C11A—N1A—C15A—C14A	57.19 (15)	C2B—C1B—C6B—C5B	−0.6 (2)
C10A—N1A—C15A—C14A	−178.85 (12)	O1B—C1B—C6B—N6B	−4.1 (3)
C13A—C14A—C15A—N1A	−56.78 (16)	C2B—C1B—C6B—N6B	177.97 (14)
O2A—C13A—C16A—C17A	5.3 (2)	O62B—N6B—C6B—C5B	−179.44 (14)
C14A—C13A—C16A—C17A	127.42 (15)	O61B—N6B—C6B—C5B	0.5 (2)
C12A—C13A—C16A—C17A	−110.78 (16)	O62B—N6B—C6B—C1B	1.9 (2)
O2A—C13A—C16A—C21A	−176.32 (14)	O61B—N6B—C6B—C1B	−178.16 (15)
C14A—C13A—C16A—C21A	−54.19 (19)		

### Hydrogen-bond geometry (Å, °)

**Table:**

*D*—H···*A*	*D*—H	H···*A*	*D*···*A*	*D*—H···*A*
O2A—H2O···O61B^i^	0.84	2.01	2.837 (2)	168
N1A—H1N···O1B	0.93	1.82	2.708 (1)	160
N1A—H1N···O62B	0.93	2.40	3.007 (2)	123
C3A—H3AA···O2A^ii^	0.95	2.47	3.338 (2)	152
C6A—H6AA···O22B^iii^	0.95	2.41	3.286 (2)	153
C8A—H8AA···O21B^iii^	0.99	2.61	3.544 (2)	158
C8A—H8AB···O61B^iv^	0.99	2.48	3.460 (2)	170
C14A—H14A···O62B^i^	0.99	2.59	3.486 (2)	150
C15A—H15A···O41B^v^	0.99	2.58	3.461 (2)	148
C18A—H18A···O22B^vi^	0.95	2.42	3.131 (2)	131

Symmetry codes: (i) *x*+1/2, −*y*+1/2, *z*; (ii) −*x*+3/2, *y*+1/2, *z*+1/2; (iii) *x*−1/2, −*y*+1/2, *z*; (iv) −*x*+3/2, *y*−1/2, *z*+1/2; (v) *x*, *y*−1, *z*; (vi) −*x*+5/2, *y*−1/2, *z*−1/2.
